# A Computational Method of Defining Potential Biomarkers based on Differential Sub-Networks

**DOI:** 10.1038/s41598-017-14682-5

**Published:** 2017-10-30

**Authors:** Xin Huang, Xiaohui Lin, Jun Zeng, Lichao Wang, Peiyuan Yin, Lina Zhou, Chunxiu Hu, Weihong Yao

**Affiliations:** 1 School of Computer Science & Technology, Dalian University of Technology, 116024 Dalian, China; 2 CAS Key Laboratory of Separation Science for Analytical Chemistry, Dalian Institute of Chemical Physics, Chinese Academy of Sciences, Dalian, 116023 China

## Abstract

Analyzing omics data from a network-based perspective can facilitate biomarker discovery. To improve disease diagnosis and identify prospective information indicating the onset of complex disease, a computational method for identifying potential biomarkers based on differential sub-networks (PB-DSN) is developed. In PB-DSN, Pearson correlation coefficient (PCC) is used to measure the relationship between feature ratios and to infer potential networks. A differential sub-network is extracted to identify crucial information for discriminating different groups and indicating the emergence of complex diseases. Subsequently, PB-DSN defines potential biomarkers based on the topological analysis of these differential sub-networks. In this study, PB-DSN is applied to handle a static genomics dataset of small, round blue cell tumors and a time-series metabolomics dataset of hepatocellular carcinoma. PB-DSN is compared with support vector machine-recursive feature elimination, multivariate empirical Bayes statistics, analyzing time-series data based on dynamic networks, molecular networks based on PCC, PinnacleZ, graph-based iterative group analysis, KeyPathwayMiner and BioNet. The better performance of PB-DSN not only demonstrates its effectiveness for the identification of discriminative features that facilitate disease classification, but also shows its potential for the identification of warning signals.

## Introduction

Biomarkers can provide information on pathogenic processes and pharmacological responses for a therapeutic intervention^1^. The identification of biomarkers for clinical diagnosis is one of several interesting topics in medical research^2^. Although measurements pertaining to discriminative molecules have been traditionally applied in the clinic, identifying meaningful biomarkers for clinical diagnostics based on information-rich biological data is challenging^3^. To identify discriminative molecules, different approaches for feature selection, such as support vector machine-recursive feature elimination (SVM-RFE)^4^, genetic algorithms (GAs)^5^ and random forests (RFs)^6^, have been widely applied^7–9^. These methods select features based on the feature expression values among different classes rather than the changes in the feature relationships. However, a feature is also important if it has a remarkable joint effect on others^10^. Since molecules interact and relate to each other, exploring changes in the relationships among molecules to obtain a comprehensive understanding of disease mechanisms has attracted increasing attention in recent years^11–14^. Hence, analyzing the biological data from a network perspective could be a better strategy for discovering key biomarkers and facilitating the study of disease phenotypes.

Disease development is usually studied from two aspects: static and dynamic. In clinical studies, static and dynamic (or time-series) data are applied to meet different clinical goals. Static data are used to compare changes under different conditions and to define the discriminative information. To extract information from static data, different network construction methods and network analysis techniques have been proposed. Pearson correlation coefficient (PCC) which measures associated relationships of features has been widely applied to construct the networks^15–17^, and the hubs are retained as key factors. Krumsiek *et al*.^18,19^ used the partial correlation coefficient to construct networks for biological data analyses. In metabolomics, a ratio could be designated as the pathway reaction in which one metabolite is converted into another metabolite via single or multiple reaction pathways^20^. Thus, Netzer *et al*.^20^ constructed a network based on the paired biomarker identifier values of the metabolite ratios. PinnacleZ^21^ applied mutual information to calculate the discriminative ability of the network. Graph-based iterative group analysis (GiGA)^22^ ranked the features in the network and identifies the informative sub-network based on the *p*-value calculated using the ranks of the features. KeyPathwayMiner^23^ applied ant colony heuristic to screen for the key sub-network. BioNet^24^ used the integer-linear programming approach to define the informative sub-network. Other efficient network-based methods exist, including a two-step module cover^25^ and condition-specific sub-networks (COSINE)^26^. Additional state-of-the-art methods have been summarized in a recent review paper^27^.

As biological processes are dynamic, the systematic exploration of the temporal responses of molecules could facilitate the extraction of potential biomarkers that indicate the onset of complex diseases^28^. The early diagnosis of complex diseases could prevent the qualitative deterioration of patients and improve survival rates. However, extracting potential biomarkers of complex diseases based on time-series data is a notable challenge. For example, the behavior of hepatocellular carcinoma (HCC) at early disease stages shows little apparent difference from that of precancerous cirrhosis (CIR)^29^. Therefore, to explore the dynamics of disease development and screen for early warning signals, some methods for analyzing time-series data have been proposed. Tai *et al*.^30^ selected important molecules using Hotelling’s T^2^, whereas Chen *et al*. calculated the composite index to identify the dynamic network biomarkers of complex diseases^31,32^. We also proposed a strategy for analyzing time-series data based on dynamic networks (ATSD-DN) to define the warning signal^33^.

In the present study, we propose a computational method that defines potential biomarkers based on differential sub-networks (PB-DSN). PB-DSN explores the changes in correlation between feature ratios among different groups to define differential sub-networks. Subsequently, the hub vertices are identified as key feature ratios to discriminate different group samples. PB-DSN can also assess changes in correlations during disease development along time points to define differential sub-networks and selects hub vertices as key information for disease phenotyping. Moreover, signals from the sub-network consisting of the edges associated with the hub vertex can be used to indicate the onset of a specific disease stage. Hence, PB-DSN can analyze both static biological data and time-series data. In this study, a static malignant tumor genomics dataset and a time-series metabolomics dataset from a rat model of DEN-induced HCC are used to validate the performance of PB-DSN.

## Results

### Application of PB-DSN in the static dataset

Many studies have explored the mechanisms of malignant tumors from the viewpoint of genomics^34–36^. A gene can signify a disease state if its expression is suppressive or augmentative under certain clinical conditions^37^. However, some diseases result from multifaceted gene webs that interact with each other in complex ways^38^.

Small, round blue cell tumors (SRBCTs) include four subtypes: neuroblastoma (NB), rhabdomyosarcoma (RMS), non-Hodgkin lymphoma (NHL) and the Ewing family of tumors (EWS). The routine histological appearances of these four tumors are similar^39^. These cancers are not distinguished well by light microscopy, and there is no single test that can precisely separate the different cancers^39^. An accurate diagnosis of the type of SRBCT is essential for providing patients with the appropriate treatment.

Training and test subsets (see supplement information) exist for four different groups of SRBCTs, including EWS, RMS, Burkitt lymphoma (BL, a subset of NHL), and NB. Detailed information about these datasets can be found in the literature^39^. PB-DSN is used to study genomic problems at a network level. Figure S1 shows the workflow of PB-DSN. A feature is retained if the |log(fold-change)| is greater than or equal to 3 between any two subtype groups. Eighty-one features are retained, and a total of 3240 ratios are computed to construct the networks. The network *G*_*EWS*_ is built based on these 3240 ratios. If *PCC* of two ratios is greater than or equal to 0.7 in EWS, then the two ratios are linked with a red edge in *G*_*EWS*_. If *PCC* of two ratios is less than or equal to −0.7 in EWS, then the edge is green in *G*_*EWS*_. *G*_*RMS*_, *G*_*BL*_ and *G*_*NB*_ are constructed using the same method applied for constructing *G*_*EWS*_.

To define the discriminative information for separating EWS from the other three groups, in this study, an edge appearing in *G*_*EWS*_ that has different behaviors in two of the other three networks (*G*_*RMS*_, *G*_*BL*_ and *G*_*NB*_) is regarded as a differential edge of EWS, and all differential edges of EWS constitute a differential sub-network of EWS (*SG*_*EWS*_). The corresponding expression of the edges in *SG*_*EWS*_ in the other three groups constitutes the sub-networks *SG*_*EWS*-*RMS*_, *SG*_*EWS*-*BL*_ and *SG*_*EWS*-*NB*_. Expanding on this idea, let *G* = (*V*(*G*), *E*(*G*)) be a graph, *V*(*G*) is the vertex set of *G* and *E*(*G*) is the edge set of *G*. (1) *SG*_*EWS*-*RMS*_ = (*V*(*SG*_*EWS-RMS*_), *E*(*SG*_*EWS*-*RMS*_)), where *V*(*SG*_*EWS*-*RMS*_) = *V*(*SG*_*EWS*_) and *E*(*SG*_*EWS*-*RMS*_) = *E*(*SG*_*EWS*_) ∩ *E*(*G*_*RMS*_); (2) *SG*_*EWS-BL*_ = (*V*(*SG*_*EWS-BL*_), *E*(*SG*_*EWS-BL*_)), where *V*(*SG*_*EWS-BL*_) = *V*(*SG*_*EWS*_) and *E*(*SG*_*EWS*-*BL*_) = *E*(*SG*_*EWS*_) ∩ *E*(*G*_*BL*_); (3) *SG*_*EWS*-*NB*_ = (*V*(*SG*_*EWS*-*NB*_), *E*(*SG*_*EWS*-*NB*_)), where *V*(*SG*_*EWS*-*NB*_) = *V*(*SG*_*EWS*_) and *E*(*SG*_*EWS*-*NB*_) = *E*(*SG*_*EWS*_) ∩ *E*(*G*_*NB*_). The edges in *SG*_*EWS-RMS*_, *SG*_*EWS-BL*_ and *SG*_*EWS*-*NB*_ have the same color as the corresponding ones in *G*_*RMS*_, *G*_*BL*_ and *G*_*NB*_, respectively.

**Figure 1:**
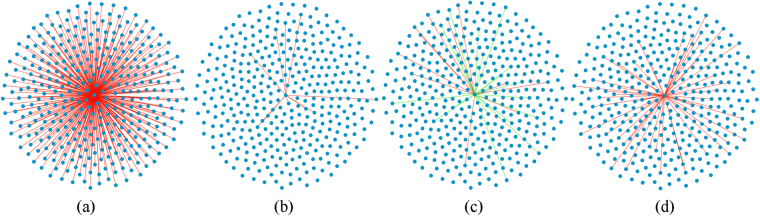
Star sub-networks based on ratio 1. (**a**–**d**) The star sub-networks consisting of the edges linked with ratio 1 in *SG*_*EWS*_, *SG*_*EWS-RMS*_, *SG*_*EWS-BL*_ and *SG*_*EWS*-*NB*_, respectively. Ratios in the four sub-networks are same. The numbers of the connections with ratio 1 in *SG*_*EWS*_, *SG*_*EWS-RMS*_, *SG*_*EWS-BL*_ and *SG*_*EWS*-*NB*_ are 370, 10, 32 and 50.

**Figure 2:**
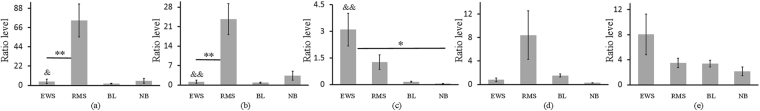
Statistical analysis of the top 5 ratios. (**a**–**e**) The statistical analysis (the mean ± the S.E.) of ratio *i* (1 ≤ *i* ≤ 5), respectively.

Subsequently, the vertices in *SG*_*EWS*_ are ranked according to their degrees in descending order. The node with the highest degree (ratio 1) is selected for further analysis. The star sub-network consisting of the edges linked to the ratio 1 in *SG*_*EWS*_ is defined and shown in Fig. 1(a). The star sub-networks consisting of the edges linked with ratio1 in *SG*_*EWS-RMS*_, *SG*_*EWS-BL*_ and *SG*_*EWS*-*NB*_ are also shown in Fig. 1. These data clearly express the difference between EWS and the other three groups and reveal that the correlations of some ratios in EWS samples are significantly different from those in the other three groups. The top 5 vertices in *SG*_*EWS*_ (see Table S1) are retained as potential biomarkers; the statistical analysis is shown in Fig. 2. The differential expression levels of ratio 1 and ratio 2 in EWS and RMS indicate that they can separate the two malignant tumor samples well. Ratio 3 is significantly decreased in NB compared with that of EWS. As the levels of ratio 1, ratio 2, and ratio 3 show significant differences between EWS and non-EWS groups, these values could be used to separate EWS from non-EWS samples. The level of ratio 4 in EWS is remarkably lower than that in RMS, thereby contributing to the discrimination of the two tumor samples. Ratio 5 increases in EWS and could be used to distinguish EWS and non-EWS samples.

PB-DSN is compared with PinnacleZ^21^, GiGA^22^, KeyPathwayMiner^23^, BioNet^24^ and the popular statistical analysis method SVM-RFE^4^. We also compare PB-DSN with molecular network based on PCC (MN-PCC) which builds the networks on features instead of feature ratios and applies the same network analysis method as PB-DSN. In PinnacleZ, KeyPathwayMiner and GiGA, to reduce the irrelevant features and improve the classification performance, the upper bound of the sub-network size is $\sqrt{N}$, where *N* is the number of total features in the network. PinnacleZ and GiGA select the sub-network with the best discriminative ability to discriminate the different diseases. The value of parameter *l* in KeyPathwayMiner is set as 0. A false-discovery rate of 10^−6^ is used in BioNet. In SVM-RFE, the kernel function is *linear* and the value of penalty factor is set as 1. In MN-PCC, if |*PCC*| of two features is great than, or equal to 0.7, then there is an edge between the two features. In PB-DSN *τ* is set as 0.7. To compare the performance of these methods, the binary logistic regression is performed. The areas under the curve (AUCs) of separated EWS and non-EWS samples are listed in Table 1. The AUCs obtained using PB-DSN, MN-PCC, PinnacleZ, GiGA, KeyPathwayMiner, BioNet, and SVM-RFE are 1.000, 0.991, 0.940, 0.953, 0.939, 0.987 and 0.789, respectively, in the training set. The corresponding AUC values in the test set are 1.000, 1.000, 0.905, 1.000, 0.786, 0.917 and 0.595, respectively.

**Table 1:** Comparison of the ROC analysis (AUC) for different methods in separating EWS from non-EWS.

Method	Training set	Test set
PB-DSN	1.000	1.000
MN-PCC	0.991	1.000
PinnacleZ	0.940	0.905
GiGA	0.953	1.000
KeyPathwayMiner	0.939	0.786
BioNet	0.987	0.917
SVM-RFE	0.789	0.595

A similar method is also used to analyze BL *vs*. non-BL, RMS *vs*. non-RMS and NB *vs*. non-NB, and the results are shown in Tables 2–4. Table 2 shows that PB-DSN has a higher AUC than PinnacleZ, KeyPathwayMiner and SVM-RFE for separating BL and non-BL samples in the training set and exhibits the same performance as MN-PCC, GiGA and BioNet. In the test set, seven methods have the same AUC values. In the case of discriminating samples between RMS and non-RMS groups (see Table 3), PB-DSN has a slightly lower performance than PinnacleZ, GiGA, BioNet and SVM-RFE in the training set, but has the same performance in the test set. Compared with MN-PCC and KeyPathwayMiner, PB-DSN has a remarkable advantage for separating samples between RMS and non-RMS groups in the training and test sets. For NB *vs*. non-NB (see Table 4), PB-DSN has a better performance than PinnacleZ, GiGA and SVM-RFE in the training set. The AUCs of PB-DSN, MN-PCC, KeyPathwayMiner and BioNet are the same in the training set. In the test set, PB-DSN, MN-PCC, PinnacleZ, GiGA, KeyPathwayMiner and BioNet can well separate the NBs from the non-NB samples, whereas the AUCs of SVM-RFE are markedly low. The performance of PB-DSN shows better potential to identify discriminative information for the improvement of disease diagnosis.

**Table 2:** Comparison of the ROC analysis (AUC) for different methods in separating BL from non-BL.

Method	Training set	Test set
PB-DSN	1.000	1.000
MN-PCC	1.000	1.000
PinnacleZ	0.998	1.000
GiGA	1.000	1.000
KeyPathwayMiner	0.964	1.000
BioNet	1.000	1.000
SVM-RFE	0.889	1.000

**Table 3:** Comparison of the ROC analysis (AUC) for different methods in separating RMS from non-RMS.

Method	Training set	Test set
PB-DSN	0.965	1.000
MN-PCC	0.753	0.867
PinnacleZ	0.985	1.000
GiGA	1.000	1.000
KeyPathwayMiner	0.866	0.853
BioNet	1.000	1.000
SVM-RFE	1.000	1.000

**Table 4:** Comparison of the ROC analysis (AUC) for different methods in separating NB from non-NB.

Method	Training set	Test set
PB-DSN	1.000	1.000
MN-PCC	1.000	1.000
PinnacleZ	0.997	1.000
GiGA	0.997	1.000
KeyPathwayMiner	1.000	1.000
BioNet	1.000	1.000
SVM-RFE	0.750	0.595

### Application of PB-DSN in the time-series dataset

Metabolomics, a powerful platform in systems biology used to study changes in holistic low-molecular-weight metabolites (≤1500 Da), plays a significant role in different fields of life science^40–42^. The dynamics of metabolite concentrations reflect physiological and pathological disturbances, and studying cancer from the perspective of cell-reprogrammed metabolism can provide insights into the process of carcinogenesis^28,43^. Thus, metabolomics studies have been successfully employed in some cases to screen for biomarkers of malignant tumors^44–46^. HCC is one of the major diseases with serious effects in humans. The early and precise diagnosis of HCC is crucial for ensuring that patients receive the appropriate treatment. However, due to the rapid development and early metastasis of HCC^47^, it is difficult to improve the performance of HCC diagnosis and, in particular, to distinguish small malignant HCCs from precancerous CIR samples. Although some traditional tumor markers (i.e., *α*-fetoprotein) are effective for HCC discrimination, the poor sensitivity of these molecules suggests that they are far from ideal^47,48^. Thus, developing efficient methods for the extraction of new biomarkers that signal HCC onset is urgently needed.

The metabolomics training set used in this study contains control and model groups and has been reported in a previous study^28^. Week 0 was defined as the starting time point of animal experiment. The collection of time-series sera set was conducted from week 8 to week 20 once every 2 weeks. The model group contains three stages: week 8 (hepatitis (H) stage, *S*_1_), week 10–14 (CIR stage, *S*_2_–*S*_4_) and week 16–20 (HCC stage, *S*_5_–*S*_7_). *S*_1_, *S*_4_, and *S*_7_ are the typical time points of the corresponding liver disease stages, whereas *S*_2_ and *S*_5_ are the first time points of the corresponding liver diseases. If a variable has missing values in a group, we replace these values with the minimum nonzero value in that group at the same time point. A |log(fold-change)| greater than or equal to 1 is used to filter the non-informative features, and seventeen features are selected based on the typical time points in three sub-problems (H *vs*. CIR, H *vs*. HCC and CIR *vs*. HCC). In total, 136 metabolite ratios are computed based on these 17 metabolites to construct the networks.

**Figure 3:**
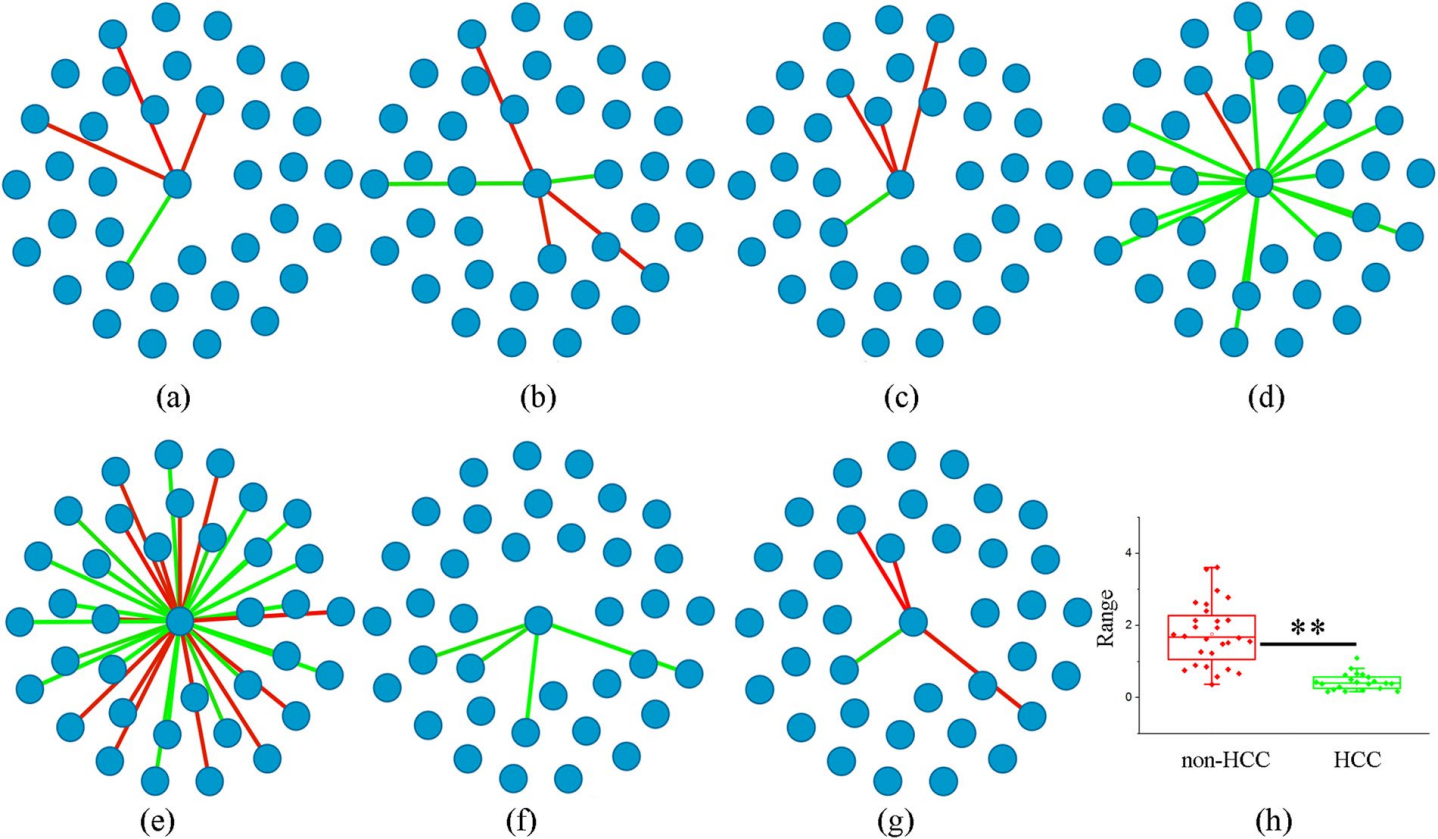
Star sub-networks based on N,N-dimethylglycine/threonic acid and box plot. (**a**–**g**) The star sub-networks consisting of the edges linked with N,N-dimethylglycine/threonic acid during disease development. Ratios in the seven sub-networks are same. The numbers of the connections with N,N-dimethylglycine/threonic acid in *SG*_*i*_ (1 ≤ *i* ≤ 7) are 4, 5, 4, 18, 36, 4 and 4. (**h**) The box plot of N,N-dimethylglycine/threonic acid.

To screen the prospective information of HCC, PB-DSN focuses on *S*_5_, which is the starting time point of HCC, and extracts differential edges to infer the differential sub-network of *S*_5_ (*SG*_5_). The differential edges are those that appear in the network of *S*_5_ but have different behaviors in most (2/3 in this study) of other networks in this time-series dataset at *S*_*t*_ (1 ≤ *t* ≤ 4). In *SG*_5_, the hub vertex (N,N-dimethylglycine/threonic acid) having the largest degree and its associated nodes are selected for further analysis. Figure 3 shows the dynamics of the correlation between N,N-dimethylglycine/threonic acid and its associated nodes in disease initiation and progression. We observed that the correlations of feature ratios change with the development of liver disease. Differences in the correlations of ratios between the starting time point of HCC and the stages prior to HCC could represent the onset of HCC. Therefore, changes in the correlations between N,N-dimethylglycine/threonic acid and its associated nodes could, at the network level, be critical information signaling the emergence of disease deterioration during liver disease development. The statistical result of N,N-dimethylglycine/threonic acid, as shown in Fig. 3(h), indicates that N,N-dimethylglycine/threonic acid is significantly different between HCC and non-HCC groups; thus, N,N-dimethylglycine/threonic acid exhibits potential for distinguishing HCC samples from non-HCC samples.

**Figure 4:**
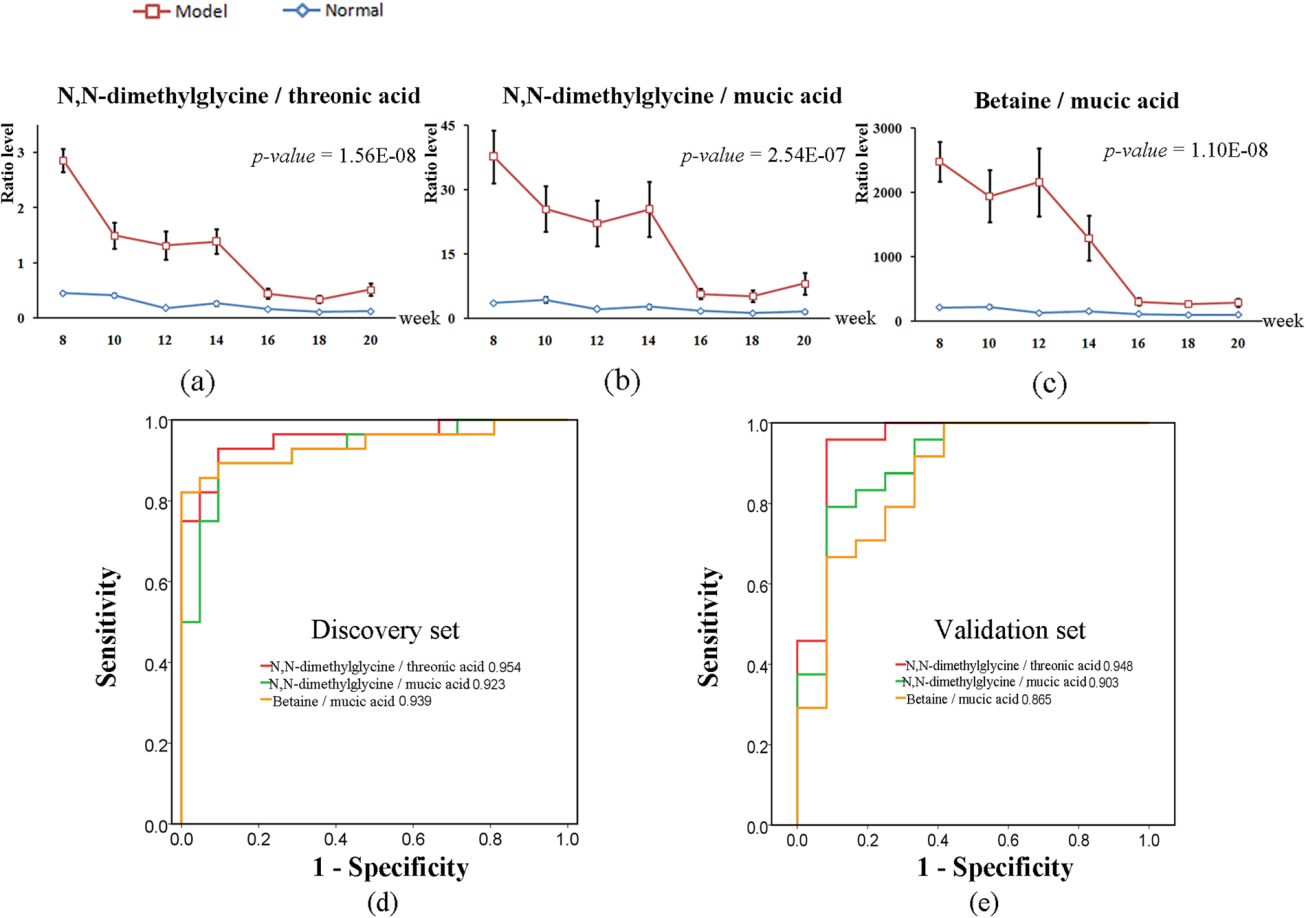
Statistical analysis of the 3 metabolite ratios. (**a**–**c**) The metabolic trajectories (the mean ± the S.E.) of N,N-dimethylglycine/threonic acid, N,N-dimethylglycine/mucic acid and betaine/mucic acid in the training set. (**d**,**e**) The ROC curves of these 3 metabolite ratios in the training and test sets.

The vertices in *SG*_5_ are ranked in descending order based on their degrees, and the top 5 ratios (see Table S2) are selected for the subsequent statistical analysis. Among the 5 ratios, the levels of 3 metabolite ratios showed significant differences between the model and age-matched groups at any time point (Table S3); thus, these metabolite ratios (N,N-dimethylglycine/mucic acid, N,N-dimethylglycine/threonic acid and betaine/mucic acid) contribute to separating the samples between control and model groups. Figure 4(a–c) show the metabolic trajectory of these 3 ratios along the time points in the training set. The significant differences of these 3 metabolite ratios are shown between HCC and non-HCC groups. Thus, we find that when the levels of these 3 metabolite ratios in non-HCC samples significantly decrease, HCC occurs. The AUCs of the 3 ratios used to discriminate HCC from non-HCC groups are 0.954, 0.923, and 0.939 in the training set, respectively (Fig. 4(d)). The detailed results of statistical analysis shown in Tables S4–S6 suggest that the levels of the 3 metabolite ratios exhibit significant differences between any time point in the HCC stage and any time point in the non-HCC stage, further indicating the ability to discriminate between HCC and non-HCC samples. Notably, for N,N-dimethylglycine/threonic acid, significant differences are also observed between the H stage and any time point in the CIR stage. The significantly decreasing level of N,N-dimethylglycine/threonic acid at different disease stages could suggest its potential for a more complete presentation of liver disease development.

In the present study, the external test set (see supplement information) contains 36 sera from 6 model rats monitored at 6 time points (i.e., *S*_1_–*S*_6_). Histological examinations to validate HCC reveal that *S*_1_–*S*_4_ are the pre-cancer stage, whereas *S*_5_–*S*_6_ are the HCC stage. The AUCs of the 3 metabolite ratios in the test set are 0.948, 0.903, and 0.865, respectively, for the separation of HCC and non-HCC samples (Fig. 4(e)).

To evaluate the performance of PB-DSN, we compared this method with multivariate empirical Bayes statistics (MEBA)^30^, ATSD-DN^33^, MN-PCC, PinnacleZ^21^, GiGA^22^, KeyPathwayMiner^23^ and BioNet^24^. In ATSD-DN, *τ* is set as 0.7, and the ratio of imidazole-4-acetic acid/trimethylamine N-oxide is selected. Trimethylamine N-oxide is selected by MN-PCC. The top ratio in PB-DSN is N,N-dimethylglycine/threonic acid. In MEBA, based on Hotelling’s T^2^, top 3 features are selected to discriminate different diseases. PinnacleZ, GiGA, KeyPathwayMiner and BioNet select the sub-network with the best discriminative ability.

The comparison results shown in Table 5 indicate that in the training set, the performance of PB-DSN for separating disease and normal groups is better than those of ATSD-DN, MN-PCC and PinnacleZ. The AUC obtained using PB-DSN for discriminating HCC and non-HCC samples is 0.954, which is higher than the AUCs of 0.808, 0.820, 0.884, 0.815, 0.900 and 0.934 obtained by ATSD-DN, MN-PCC, PinnacleZ, GiGA, KeyPathwayMiner and BioNet, respectively. The performance of PB-DSN is only 0.002 lower than that of MEBA. In the test set, the AUC obtained using PB-DSN is better than those of other methods except ATSD-DN and KeyPathwayMiner. To discriminate H from the CIR samples, the AUCs obtained for the training set by PB-DSN, MEBA, ATSD-DN, MN-PCC, PinnacleZ, GiGA, KeyPathwayMiner and BioNet are 0.966, 1.000, 0.776, 0.619, 0.912, 0.687, 0.966 and 0.959, respectively, and the corresponding AUCs in the test set are 0.972, 0.917, 0.870, 0.741, 0.787, 0.750, 0.907 and 0.889, respectively. In our previous study^28^, a ratio of creatine/betaine was identified. The AUCs of creatine/betaine in the training and test sets are shown in Table 5. In all cases, the AUCs of N,N-dimethylglycine/threonic acid selected by PB-DSN are superior to those of creatine/betaine. The better performance of PB-DSN for analyzing the time-series dataset illustrates the potential of this method to define prospective signals that indicates the onset of HCC, thereby improving the precise diagnoses of different liver diseases.

**Table 5:** Comparison of the ROC analysis (AUC) in metabolomics data.

Method	N *vs*. M	HCC *vs*. non-HCC		H *vs*. CIR	
	Training set	Training set	Test set	Training set	Test set
PB-DSN	0.898	0.954	0.948	0.966	0.972
MEBA	0.987	0.956	0.903	1.000	0.917
ATSD-DN	0.699	0.808	0.965	0.776	0.870
MN-PCC	0.567	0.820	0.913	0.619	0.741
PinnacleZ	0.890	0.884	0.701	0.912	0.787
GiGA	0.910	0.815	0.813	0.687	0.750
KeyPathwayMiner	0.915	0.900	0.958	0.966	0.907
BioNet	0.915	0.934	0.917	0.959	0.889
Creatine/betaine	0.860	0.905	0.792	0.660	0.667

## Discussion

The precise, early diagnosis of malignant tumors can better facilitate appropriate treatments and improve the survival rates of patients. However, due to the complex factors of individual differences, epigenetics and environmental effects, identifying efficient biomarkers remains a challenge. Molecules interact with each other in networks or pathways to implement biological functions^49^. In contrast to the molecule level, deregulation at the pathway level is more critical to carcinogenesis^50^. Thus, discovering biomarkers from a network-based perspective can provide a more efficient strategy to characterize disease phenotypes.

To define discriminative information for classifying different disease groups in the static dataset and to identify the warning signals of disease in the time-series dataset, PB-DSN examines changes in the relationships among ratios and extracts differential edges to infer differential sub-networks.

To identify discriminative information in the static dataset about a specific group, the differential edges with different behaviors between the network of the specific group and most of other networks are extracted. Based on these differential edges, the differential sub-network, which reflects the discriminative information between the specific group and other groups, is constructed. The vertices with large degree in the differential sub-network contain crucial information, as their relationships with many other feature ratios (i.e., adjacent vertices) have changed and, thus, the correlations of the feature ratios are much close in the specific group.

Exploring the important prospective information about the onset of a specific physiological or pathological stage is crucial. To define prospective information about the disease severity and phenotype based on the time-series dataset, PB-DSN focuses on a specific time point (e.g., the starting time point of HCC) and traces the changes in relationships of the ratios from the beginning of the assessment to the specific time point. Subsequently, PB-DSN extracts the differential edges that have different behaviors between the network of the specific time point and most other networks of the earlier time points. Based on these differential edges, a differential sub-network, which reflects the discriminative information between the specific time point and the before time points, is constructed. The vertices with the large degree in the differential sub-network contain crucial prospective information about the onset of a specific physiological or pathological phenomenon, as their relationships with many other feature ratios (i.e., adjacent vertices) have changed; thus, the correlation of feature ratios is much close at the specific time point.

Based on the comparisons in the analysis of static datasets, PB-DSN outperforms MN-PCC, PinnacleZ, GiGA, KeyPathwayMiner, BioNet and SVM-RFE. Hence, by studying the differences of the feature ratio correlations among different groups, PB-DSN can be used to mine important discriminative information.

In the time-series metabolomics data, three metabolite ratios, N,N-dimethylglycine/threonic acid, N,N-dimethylglycine/mucic acid and betaine/mucic acid, are defined. Increased N,N-dimethylglycine is considered an important indicator for a shift of homocysteine remethylation towards the betaine-homocysteine-methyltransferase reaction in liver CIR^51^. Betaine is an important methyl donor that plays a significant role in hepatic methyl balance^28^. These two compounds are closely associated with homocysteine remethylation. Moreover, threonic acid is a product of ascorbic acid oxidation^52^ and is thus associated with systemic oxidative stress in patients. In the present study, mucic acid is decreased in the model group compared with controls, which likely indicates that low levels of mucic acid are associated with the onset of liver disease. The combinations of N,N-dimethylglycine/threonic acid, N,N-dimethylglycine/mucic acid and betaine/mucic acid as biomarkers may improve the diagnosis of HCC development. These three ratios can promote the discrimination of metabolic differences, as the combination patterns indicate that different physiological perspectives are considered based on different metabolic pathways rather than traditional individual features or single pathway-derived metabolites. Larger HCC patient cohorts are needed to validate these results in future studies.

PB-DSN is compared with MEBA^30^, ATSD-DN^33^, MN-PCC, PinnacleZ^21^, GiGA^22^, KeyPathwayMiner^23^ and BioNet^24^ in the analysis of time-series data. The performance of PB-DSN is better than those of other methods, except MEBA for discriminating between non-HCC and HCC samples in the training set. In the test set, the biomarker performance indicated by PB-DSN is also efficient. In the case of separating samples between H and CIR groups, PB-DSN only has the slightly lower performance than that of MEBA in the training set, but has the best performance in the test set. The biomarkers identified by PB-DSN are effective in discriminating diseases from normal control samples. The better performance shows that compared with other methods, PB-DSN is more advantageous for extracting prospective information to facilitate the diagnosis of HCC.

Moreover, for PB-DSN and some compared methods, we made experiments to show the influence of the parameters on the effectiveness. Different parameter settings are tested and the corresponding performances are given in Tables S7–S14.

In summary, PB-DSN analyzes biological data from networks to define discriminative information and prospective signals of complex diseases. The application of PB-DSN in the two malignant tumor datasets shows that this method has the potential to effectively define discriminative information from a static dataset and to identify the prospective signals from a time-series dataset. Moreover, the network analysis method of PB-DSN can be applied in molecular networks, which also have the effective performance in some cases.

## Methods

Let $D=\{{S}_{t}|1\le t\le {N}_{s}\}$ represent the data set, where *S*_*t*_ is a data subset of the *t*th disease group in a static problem or a data subset of the *t*th time point in the dynamic problem and *N*_*s*_ represents the number of the various disease groups or the number of time points. Let $F=\{{f}_{1},\,{f}_{2},\,\ldots ,\,{f}_{m}\}$ represent the feature set, where *m* represents the number of features. Since a ratio can be designated as the pathway reaction in which one metabolite is converted into another metabolite via single or multiple reaction pathways, the relationship of these ratios was explored to construct the network used to analyze the metabolomics data^20^. Furthermore, ratios of gene expression levels were also studied in genomics^53,54^. Hence, PB-DSN also studies feature ratios to analyze the biological data.

In PB-DSN, the feature ratio that means the ratio of feature concentration or expression level is defined as *r*_*ij*_(*t*) = *f*_*it*_/*f*_*jt*_, where *f*_*it*_ is *f*_*i*_ at *S*_*t*_ (1 ≤ *i* < *j* ≤ *m*, 1 ≤ *t* ≤ *N*_*s*_).

### Network construction

The Pearson correlation coefficient of two feature ratios *r*_*x*_(*t*) and *r*_*y*_(*t*) in group (or at time point) *S*_*t*_ (1 ≤ *t* ≤ *N*_*s*_) is defined as

1 $$PCC({r}_{x}(t),{r}_{y}(t))=\frac{1}{{n}_{t}-1}\sum _{k=1}^{{n}_{t}}(\frac{{r}_{x}(t,k)-{\mu }_{{r}_{x}(t)}}{{\sigma }_{{r}_{x}(t)}})(\frac{{r}_{y}(t,k)-{\mu }_{{r}_{y}(t)}}{{\sigma }_{{r}_{y}(t)}}),$$

where *r*_*x*_(*t*, *k*) and *r*_*y*_(*t*, *k*) are the values of ratio *r*_*x*_(*t*) and *r*_*y*_(*t*) in the *k*th sample at *S*_*t*_, ${\mu }_{{r}_{x}(t)}$ and ${\mu }_{{r}_{y}(t)}$ are the means of ratio *r*_*x*_(*t*) and *r*_*y*_(*t*), ${\sigma }_{{r}_{x}(t)}$ and ${\sigma }_{{r}_{y}(t)}$ are the standard deviation, and *n*_*t*_ is the number of the samples at *S*_*t*_. The Pearson correlation coefficient describes the relationships between variables in a phenomenological form. When two variables occur adjacently in a pathway or are derived from a common precursor, the correlation coefficient is positive, and when one variable is used to directly or indirectly generate the other, the correlation coefficient is negtive^55^. Large |*PCC*(*r*_*x*_(*t*), *r*_*y*_(*t*))| suggests that the two corresponding feature ratios are closely related to each other at *S*_*t*_. Hence, the network *G*_*t*_ is built based on the Pearson correlation coefficient for depicting the relationships among feature ratios at *S*_*t*_. Let each feature ratio represent a vertex in the network, and when the Pearson correlation coefficient of the two feature ratios |*PCC*(*r*_*x*_(*t*), *r*_*y*_(*t*))| ≥ *τ*, there is an edge between the two corresponding feature ratios *r*_*x*_(*t*) and *r*_*y*_(*t*) in *G*_*t*_. Since the Pearson correlation coefficient, which represents the different relationships of the two ratios, may be positive or negative, the edge is colored red for* PCC*(*r*_*x*_(*t*), *r*_*y*_(*t*)) ≥ *τ* and green for *PCC*(*r*_*x*_(*t*), *r*_*y*_(*t*)) ≤ *-τ*.

### Defining the differential sub-network

In a complex biological system, the relationship of the feature ratios in different physiological or pathological phenomena may be different. Thus, the correlation difference among the different sample groups or along the time points could reflect different physiological or pathological changes.

**Definition 1**. Let *D* = {*S*_*t*_| 1 ≤ *t* ≤ *N*_*s*_} represent the data set, where *S*_*t*_ is a data subset of the *t*th disease group in a static problem. Let *G*_*t*_ represent the network at *S*_*t*_. If *e* ∈ *V*(*G*_*t*_) has different behaviors (i.e., disappears or has a different color) in most of the other networks, then *e* is a “differential edge” at *S*_*t*_. The sub-network, *SG*_*t*_, consisting of all the differential edges at *S*_*t*_ is called the differential sub-network at *S*_*t*_ in the static problem.

**Definition 2**. Let *D* = {*S*_*t*_| 1 ≤ *t* ≤ *N*_*s*_} represent the data set, where *S*_*t*_ is a data subset of the *t*th time point in the dynamic problems. Let *G*_*t*_ represent the network at *S*_*t*_. If *e* ∈ *V*(*G*_*t*_) has different behaviors (i.e., disappears or has a different color) in most of the networks *G*_*p*_ (1 ≤ *p* < *t*), then *e* is a “differential signal edge” of *S*_*t*_. The sub-network, *SG*_*t*_, consisting of all the differential signal edges of *S*_*t*_ is called the differential sub-network at *S*_*t*_ in time-series problems.

Hence, the differential sub-network at *S*_*t*_ in a static problem contains information to discriminate group *S*_*t*_ from other groups based on the changes in the relationships between the ratios. Moreover, the differential sub-network of *S*_*t*_ in a dynamic problem contains information that could signal the onset of the specific physiological or pathological stage at *S*_*t*_, which contains certain information that is markedly different from that of the previous time point.

PB-DSN applies topological structure analysis to select the most important ratios from the differential sub-network. This method ranks the nodes of the differential sub-network at *S*_*t*_ in a descending order according to their degrees, and the top *k* ≥ 1 nodes are selected.

### The compared methods

#### SVM-RFE

This method selects the important feature subset by sequential backward elimination^4^. In each iteration, SVM-RFE measures the weights of the features based on the contribution to the hyperplane, and the features with the lowest weights are removed from the current feature subset. During the recursive procession, the feature subset with the best classification performance is retained as the selected feature subset.

#### MEBA

This method is based on multivariate empirical Bayes statistics and uses Hotelling’s T^2^ to measure the importance of features in a systematic time dimension^30^. To reduce the false positive and false negative results, MEBA applies the time-course mean profiles to evaluate treatment differences.

#### MN-PCC

*PCC* is applied to measure the relationship of the molecules and construct the networks. Subsequently, the same network analysis method as PB-DSN is applied to identify the key factors.

#### ATSD-DN

This method constructs networks based on the non-overlapping ratios of the feature ratios^33^. Two network analysis techniques, dynamic concentration analysis and topological structure analysis, are performed to identify the informative feature ratios. Among the feature ratios selected by both two techniques, the ratio with the highest AUC is considered a potential biomarker.

#### PinnacleZ

This method uses mutual information to define the discriminative ability of a sub-network^21^. To search a sub-network, PinnacleZ starts from each node in the network and performs a greedy search. During the search, PinnacleZ iteratively adds a node that is associated with other nodes in the current sub-network and can yield a maximal score increase in the sub-network. This procedure is continued until the improvement rate of the score is less than or equal to 0.05.

#### GiGA

This method selects the discriminative sub-network based on *p*-values^22^. First, GiGA assigns a rank to each feature based on expression changes and identifies the local minimum (i.e., the node with a lower rank than all direct neighbors in the network). Subsequently, each local minimum is viewed as a seed and is iteratively extended. In each iteration, the neighboring node with the smallest rank is added. After *n* steps, a sub-network with *n* nodes with a maximum rank *m* is built. Based on *p*-value, the sub-network is scored as

2 $$p=\underset{i=0}{\overset{n-1}{{\rm{\Pi }}}}\frac{m-i}{N-i},$$

where *N* is the number of total nodes in the network.

#### KeyPathwayMiner

The goal of this method is also to define the informative sub-network^23^. A strategy called Global Node Exceptions is used in KeyPathwayMiner. Moreover, ant colony heuristic is used for finding the solution to subnet problem.

#### BioNet

BioNet is an effective method that can compute provably optimal or sub-optimal solutions to screen for the maximal-scoring sub-network^24^. First, BioNet calculates the maximum likelihood score for each features based on the beta uniform mixture distribution of the *p*-values. Subsequently, integer-linear programming is applied to define the optimal sub-network in reasonable computation time.

## Electronic supplementary material

Supplementary Information

## References

[1] Atkinson AJ (2001). Biomarkers and surrogate endpoints: preferred definitions and conceptual framework. Clin. Pharmacol. Ther..

[2] Liu R, Wang X, Aihara K, Chen L (2014). Early diagnosis of complex diseases by molecular biomarkers, network biomarkers, and dynamical network biomarkers. Med. Res. Rev..

[3] Saccenti E, Hoefsloot HCJ, Smilde AK, Westerhuis JA, Hendriks MMWB (2013). Reflections on univariate and multivariate analysis of metabolomics data. Metabolomics.

[4] Guyon I, Weston J, Barnhill S, Vapnik V (2002). Gene selection for cancer classification using support vector machines. MLear..

[5] Goldberg DE, Holland JH (1988). Genetic algorithms and machine learning. MLear..

[6] Breiman L (2001). Random forests. MLear..

[7] Tapia E, Bulacio P, Angelone L (2012). Sparse and stable gene selection with consensus SVM-RFE. Pattern Recog. Lett..

[8] Diaz-Uriarte, R. & A de Andres, S. Gene selection and classification of microarray data using random forest. *BMC Bioinformatics* **7**, doi:10.1186/1471-2105-7-3 (2006). 10.1186/1471-2105-7-3 PMC1363357 16398926

[9] Li L (2005). A robust hybrid between genetic algorithm and support vector machine for extracting an optimal feature gene subset. Genomics.

[10] Chen, Y., Wang, L., Li, L., Zhang, H. & Yuan, Z. Informative gene selection and the direct classification of tumors based on relative simplicity. *BMC Bioinformatics* **17**, 10.1186/s12859-016-0893-0 (2016). 10.1186/s12859-016-0893-0 PMC4721022 26792270

[11] Long, F., Su, J. H., Liang, B., Su, L. L. & Jiang, S. J. Identification of gene biomarkers for distinguishing small-cell lung cancer from non-small-cell lung cancer using a network-based approach. *Biomed. Res. Int*., 10.1155/2015/685303 (2015). 10.1155/2015/685303 PMC4531169 26290870

[12] Feng L (2016). A network-based method for identifying prognostic gene modules in lung squamous carcinoma. Oncotarget.

[13] Nai, W. Q. *et al*. Identification of novel genes and pathways in carotid atheroma using integrated bioinformatic methods. *Sci. Rep*. **6**, 10.1038/srep18764 (2016). 10.1038/srep18764 PMC4705461 26742467

[14] Qin, C., Sun, Y. Q. & Dong, Y. D. A new method for identifying essential proteins based on network topology properties and protein complexes. *PloS One* **11**, 10.1371/journal.pone.0161042 (2016). 10.1371/journal.pone.0161042 PMC4987049 27529423

[15] Zhang X, Yang H, Gong B, Jiang C, Yang L (2012). Combined gene expression and protein interaction analysis of dynamic modularity in glioma prognosis. J. Neurooncol..

[16] Xue, H. *et al*. A modular network model of aging. *Mol. Syst. Biol*. **3**, doi:10.1038/msb4100189 (2007). 10.1038/msb4100189 PMC2174624 18059442

[17] Shao T (2015). Identification of module biomarkers from the dysregulated ceRNA-ceRNA interaction network in lung adenocarcinoma. Mol Biosyst.

[18] Krumsiek, J., Suhre, K., Illig, T., Adamski, J. & Theis, F. J. Gaussian graphical modeling reconstructs pathway reactions from high-throughput metabolomics data. *BMC Syst. Biol*. **5**, 10.1186/1752-0509-5-21 (2011). 10.1186/1752-0509-5-21 PMC3224437 21281499

[19] Castro C (2013). A study of Caenorhabditis elegans DAF-2 mutants by metabolomics and differential correlation networks. Mol. BioSyst..

[20] Netzer, M. *et al*. Profiling the human response to physical exercise: a computational strategy for the identification and kinetic analysis of metabolic biomarkers. *J. Clin. Bioinformatics* **1**, 10.1186/2043-9113-1-34 (2011). 10.1186/2043-9113-1-34 PMC3320562 22182709

[21] Chuang, H., Lee, E., Liu, Y., Lee, D. & Ideker, T. Network-based classification of breast cancer metastasis. *Mol. Syst. Biol*. **3**, 10.1038/msb4100180 (2007). 10.1038/msb4100180 PMC2063581 17940530

[22] Breitling, R., Amtmann, A. & Herzyk, P. Graph-based iterative group analysis enhances microarray interpretation. *BMC Bioinformatics* **5**, 10.1186/1471-2105-5-100 (2004). 10.1186/1471-2105-5-100 PMC509016 15272936

[23] Alcaraz, N. *et al*. KeyPathwayMiner 4.0: condition-specific pathway analysis by combining multiple omics studies and networks with Cytoscape. *BMC Syst. Biol*. **8**, 10.1186/s12918-014-0099-x (2014). 10.1186/s12918-014-0099-x PMC4236746 25134827

[24] Dittrich M, Klau G, Rosenwald A, Dandekar T, Muller T (2008). Identifying functional modules in protein-protein interaction networks: an integrated exact approach. Bioinformatics.

[25] Kim, Y., Salari, R., Wuchty, S. & Przytycka, T. Module cover - a new approach to genotype-phenotype studies. *Pac. Symp. Biocomput*, 135–146 (2013). PMC3595055 23424119

[26] Ma H, Schadt E, Kaplan L, Zhao H (2011). COSINE: condition-specific sub-network identification using a global optimization method. Bioinformatics.

[27] Batra, R. *et al*. On the performance of de novo pathway enrichment. *Syst. Biol. Appl*. **3**, 10.1038/s41540-017-0007-2 (2017). 10.1038/s41540-017-0007-2 PMC5445589 28649433

[28] Zeng, J. *et al*. Metabolomics identifies biomarker pattern for early diagnosis of hepatocellular carcinoma: from diethylnitrosamine treated rats to patients. *Sci. Rep*. **5**, 10.1038/srep16101 (2015). 10.1038/srep16101 PMC4630653 26526930

[29] Zhou L (2012). Serum metabolomics reveals the deregulation of fatty acids metabolism in hepatocellular carcinoma and chronic liver diseases. Anal. Bioanal. Chem..

[30] Tai Y, Speed T (2006). A multivariate empirical Bayes statistic for replicated microarray time course data. Ann. Stat..

[31] Chen, L., Liu, R., Liu, Z. P., Li, M. & Aihara, K. Detecting early-warning signals for sudden deterioration of complex diseases by dynamical network biomarkers. *Sci. Rep*. **2**, 10.1038/srep00342 (2012). 10.1038/srep00342 PMC3314989 22461973

[32] Li M, Zeng T, Liu R, Chen L (2014). Detecting tissue-specific early warning signals for complex diseases based on dynamical network biomarkers: study of type 2 diabetes by cross-tissue analysis. Brief Bioinform..

[33] Huang, X. *et al*. A new strategy for analyzing time-series data using dynamic networks: identifying prospective biomarkers of hepatocellular carcinoma. *Sci. Rep*. **6**, 10.1038/srep32448 (2016). 10.1038/srep32448 PMC5006023 27578360

[34] Konopka T, Nijman S (2015). Comparison of genetic variants in matched samples using thesaurus annotation. Bioinformatics.

[35] Geman, D., d’Avignon, C., Naiman, D. Q. & Winslow, R. L. Classifying gene expression profiles from pairwise mRNA comparisons. *Stat. Appl. Genet. Mol. Biol*. **3** (2004). 10.2202/1544-6115.1071 PMC1989150 16646797

[36] Yazdani A, Dunson DB (2015). A hybrid bayesian approach for genome-wide association studies on related individuals. Bioinformatics.

[37] Gibbons GH (2004). Genetic markers: progress and potential for cardiovascular disease. Circulation.

[38] Rather RA, Dhawan V (2016). Genetic markers: potential candidates for cardiovascular disease. Int. J. Cardiol..

[39] Khan J (2001). Classification and diagnostic prediction of cancers using gene expression profiling and artificial neural networks. Nat. Med..

[40] Feng, Q. *et al*. Integrated metabolomics and metagenomics analysis of plasma and urine identified microbial metabolites associated with coronary heart disease. *Sci. Rep*. **6**, 10.1038/srep22525 (2016). 10.1038/srep22525 PMC4773756 26932197

[41] Liu, P., Qi, C. B., Zhu, Q. F., Yuan, B. F. & Feng, Y. Q. Determination of thiol metabolites in human urine by stable isotope labeling in combination with pseudo-targeted mass spectrometry analysis. *Sci. Rep*. **6**, 10.1038/srep21433 (2016). 10.1038/srep21433 PMC4757830 26888486

[42] Moreno-Navarrete JM (2016). Metabolomics uncovers the role of adipose tissue PDXK in adipogenesis and systemic insulin sensitivity. Diabetologia.

[43] Jain M (2012). Metabolite profiling identifies a key role for glycine in rapid cancer cell proliferation. Science.

[44] Chan AW (2016). 1)H-NMR urinary metabolomic profiling for diagnosis of gastric cancer. Br. J. Cancer.

[45] Ke, C. *et al*. Metabolic phenotyping for monitoring ovarian cancer patients. *Sci. Rep*. **6**, 10.1038/srep23334 (2016). 10.1038/srep23334 PMC4800393 26996990

[46] Lu Y (2015). Identification of serum biomarkers associated with hepatitis B virus-related hepatocellular carcinoma and liver cirrhosis using mass-spectrometry-based metabolomics. Metabolomics.

[47] Zeng J (2014). Metabolomics study of hepatocellular carcinoma: discovery and validation of serum potential biomarkers by using capillary electrophoresis-mass spectrometry. J. Proteome Res..

[48] Parikh S, Hyman D (2007). Hepatocellular cancer: a guide for the internist. Am. J. Med..

[49] Barabasi AL, Oltvai ZN (2004). Network biology: understanding the cell’s functional organization. Nat. Rev. Genet..

[50] Chopra, P., Lee, J., Kang, J. & Lee, S. Improving cancer classification accuracy using gene pairs. *PloS One* **5**, 10.1371/journal.pone.0014305 (2010). 10.1371/journal.pone.0014305 PMC3006158 21200431

[51] Look MP (2000). Is the increase in serum cystathionine levels in patients with liver cirrhosis a consequence of impaired homocysteine transsulfuration at the level of gamma-cystathionase?. Scand. J. Gastroenterol.

[52] Isbell HS, Frush HL (1979). Oxidation of L-ascorbic acid by hydrogen peroxide: preparation of L-threonic acid. Carbohydr. Res..

[53] Netzer, M. *et al*. A coupled three-step network-based approach to identify genes associated with breast cancer. The Fourth International Conference on Bioinformatics, Biocomputational Systems and Biotechnologies, St. Maarten, Netherlands Antilles. IARIA XPS Press. (2012, March 25–30).

[54] Fang X, Netzer M, Baumgartner C, Bai C, Wang X (2013). Genetic network and gene set enrichment analysis to identify biomarkers related to cigarette smoking and lung cancer. Cancer Treat. Rev..

[55] Wang L (2015). Reconstruction and analysis of correlation networks based on GC-MS metabolomics data for young hypertensive men. Anal. Chim. Acta..

