# Supplementary material for: A Computational Method of Defining Potential Biomarkers based on Differential Sub-Networks
Source: Sci Rep. 2017 Oct 30;7:14339. doi: 10.1038/s41598-017-14682-5 (PMC5662748; doi:10.1038/s41598-017-14682-5)
Supplement: Supplementary file 1 — Supplementary Information [file 41598_2017_14682_MOESM1_ESM.pdf]

A Computational Method of Defining Potential Biomarkers based on Differential Sub-Networks

Xin Huang[a], Xiaohui Lin[a]*, Jun Zeng[b], Lichao Wang[b], Peiyuan Yin[b], Lina Zhou[b], Chunxiu Hu[b], Weihong Yao[a]

[a] School of Computer Science & Technology, Dalian University of Technology, 116024 Dalian, China.
[b] CAS Key Laboratory of Separation Science for Analytical Chemistry, Dalian Institute of Chemical Physics, Chinese Academy of Sciences, Dalian 116023, China.

* Corresponding person:
Prof. Xiaohui Lin, School of Computer Science & Technology, Dalian University of Technology, 116024 Dalian, China.
E-mail: datas@dlut.edu.cn.

The descriptions of the datasets and the definitions of training and test set

For the gene expression data, the 63 training samples included both tumor biopsy material (13 EWS and 10 RMS) and cell lines (10 EWS, 10 RMS, 12 NB and 8 Burkitt lymphomas (BL; a subset of NHL)). The test samples contained both tumors (5 EWS, 5 RMS and 4 NB) and cell lines (1 EWS, 2NB and 3 BL). Filtering for a minimal level of expression reduced the number of genes to 2308 [1].

For the metabolomics training data [2], week 0 was defined as the starting time point of the experiment. The collection of time-serial sera set was conducted from week 8 to week 20 once every 2 weeks. The serial progression of hepato-carcinogenesis in the model group was divided into three stages: week 8 (hepatitis (H) stage, S_1), week 10-14 (CIR stage, S_2-S_4) and week 16-20 (HCC stage, S_5-S_7) according to histological examination. S_1, S_4, and S_7 were the typical time points of the corresponding liver disease stages, whereas S_2 and S_5 were the first time points of the corresponding liver disease stages. The time-serial sera training set, including 7 rats from model group and 10 rats from control group. In the test set, there were 36 sera from another 6 model rats. These 6 rats were sacrificed for histological examination with the affirmance of HCC at week 18. Therefore, their sera were collected from 6 monitoring time points (i.e., S_1–S_6). Histological examinations to validate HCC reveal that S_1–S_4 were the pre-cancer stage, whereas S_5–S_6 were the HCC stage.

In many areas of information science, finding classifying or predictive relationships from data is a very important task. Initial discovery of relationships is usually done with a training set while a test set is used for evaluating whether the discovered relationships hold. More formally, a training set is a set of data used to discover potentially classifying or predictive relationships. A test set is a set of data used to assess the strength and utility of a classifying or predictive relationship.

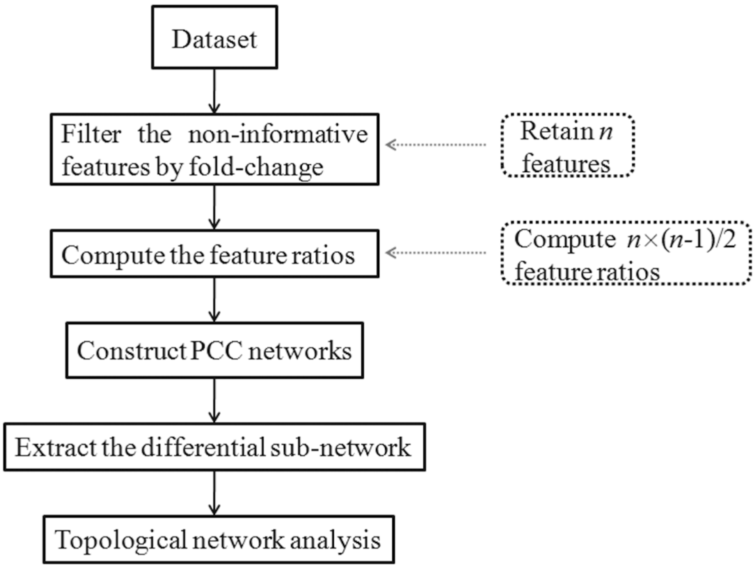

Fig. S1 The workflow of PB-DSN

Table S1 The top five ratios based on the degrees in SG_{EWS}

Node	Numerator	Denominator	Degree
Ratio 1	f_{509}	f_{2199}	370
Ratio 2	f_{187}	f_{417}	367
Ratio 3	f_{1803}	f_{2050}	365
Ratio 4	f_{1975}	f_{1980}	365
Ratio 5	f_{831}	f_{2235}	363

Table S2 The top five ratios based on the degrees in SG_5

Numerator	Denominator	Degree
N,N-dimethylglycine	Threonic acid	36
N,N-dimethylglycine	Mucic acid	33
3-Hydroxybutyric acid	Ethanolamine phosphate	33
Betaine	Mucic acid	32
Mucic acid	Imidazole-4-acetic acid	32

Table S3 The significance test of the 5 identified metabolite ratios between control and age-matched model groups

Metabolite 1 (Numerator)	Metabolite 2 (Denominator)	*p*-value						
		C8 *vs*. M8	C10 *vs*. M10	C12 *vs*. M12	C14 *vs*. M14	C16 *vs*. M16	C18 *vs*. M18	C20 *vs*. M20
N,N-dimethylglycine	Threonic acid	8.57E-10	8.30E-05	7.33E-05	4.27E-05	1.98E-03	4.36E-04	6.67E-04
N,N-dimethylglycine	Mucic acid	6.74E-06	2.63E-04	3.70E-04	6.62E-04	1.53E-03	3.25E-03	7.83E-03
3-Hydroxybutyric acid	Ethanolamine phosphate	1.02E-05	9.34E-04	2.09E-01	1.29E-01	5.53E-01	5.45E-01	7.23E-01
Betaine	Mucic acid	2.28E-07	1.36E-04	2.89E-04	1.37E-03	2.34E-03	8.09E-04	2.68E-03
Mucic acid	Imidazole-4-acetic acid	6.37E-03	1.78E-01	2.37E-03	2.53E-02	1.89E-02	1.54E-03	1.54E-02

Table S4 The significance test of N,N-dimethylglycine/threonic acid between two time points in different stages of liver disease

Time point	*p-value*					
	M10	M12	M14	M16	M18	M20
M8	1.37E-05	5.96E-04	3.10E-03	3.65E-05	8.12E-06	2.83E-05
M10	NA	NA	NA	5.83E-03	1.27E-03	1.79E-03
M12	NA	NA	NA	1.03E-02	2.63E-03	2.29E-02
M14	NA	NA	NA	3.52E-03	1.78E-03	3.93E-03

Table S5 The significance test of N,N-dimethylglycine/mucic acid between two time points in different stages of liver disease

Time point	*p-value*					
	M10	M12	M14	M16	M18	M20
M8	3.72E-03	5.08E-02	1.34E-01	2.02E-03	1.18E-03	1.27E-03
M10	NA	NA	NA	7.94E-03	3.69E-03	6.23E-03
M12	NA	NA	NA	1.57E-02	7.65E-03	3.32E-02
M14	NA	NA	NA	1.49E-02	8.47E-03	2.68E-02

Table S6 The significance test of betaine/mucic acid between two time points in different stages of liver disease

Time point	*p-value*					
	M10	M12	M14	M16	M18	M20
M8	6.46E-02	5.50E-01	7.75E-03	5.87E-04	2.05E-04	2.48E-04
M10	NA	NA	NA	8.84E-03	3.97E-03	5.67E-03
M12	NA	NA	NA	1.17E-02	8.42E-03	1.11E-02
M14	NA	NA	NA	2.98E-02	1.89E-02	2.75E-02

Table S7 The comparison between PN-DSN and BioNet for the different thresholds of false discovery rates (FDR) on the static dataset

Method	Parameter setting	EWS *vs*. non-EWS		BL *vs*. non-BL		RMS *vs*. non-RMS		NB *vs*. non-NB	
		Training set	Test set	Training set	Test set	Training set	Test set	Training set	Test set
PB-DSN		1.000	1.000	1.000	1.000	0.965	1.000	1.000	1.000
BioNet	E-07	0.987	0.917	1.000	1.000	0.980	1.000	1.000	1.000
	5E-07	0.987	0.917	1.000	1.000	1.000	1.000	1.000	1.000
	**E-06**	**0.987**	**0.917**	**1.000**	**1.000**	**1.000**	**1.000**	**1.000**	**1.000**

Bold: The parameter setting used in the manuscript.

Table S8 The comparison between PN-DSN and SVM-RFE with different kernel functions and different values of *penalty factor* on the static dataset

Method	Parameter setting	EWS *vs*. non-EWS		BL *vs*. non-BL		RMS *vs*. non-RMS		NB *vs*. non-NB	
		Training set	Test set	Training set	Test set	Training set	Test set	Training set	Test set
PB-DSN		1.000	1.000	1.000	1.000	0.965	1.000	1.000	1.000
SVM-RFE	**Linear, 1**	**0.789**	**0.595**	**0.889**	**1.000**	**1.000**	**1.000**	**0.750**	**0.595**
	Linear, 10	0.789	0.595	0.889	1.000	1.000	1.000	0.750	0.595
	RBF, 1	0.789	0.595	0.889	1.000	1.000	1.000	0.750	0.595
	RBF, 10	0.789	0.595	0.889	1.000	1.000	1.000	0.750	0.595

Bold: The parameter setting used in the manuscript. Linear: linear kernel function. RBF: radial base kernel function.

Table S9 The comparison between PN-DSN and MEBA for different top *k* features on the time-series dataset

Method	*k*	N *vs*. M	HCC *vs*. non-HCC		H *vs*. CIR	
		Training set	Training set	Test set	Training set	Test set
PB-DSN		0.898	0.954	0.948	0.966	0.972
MEBA	top 1	0.826	0.934	0.917	0.912	0.787
	top 2	0.901	0.952	0.913	0.898	0.843
	**top 3**	**0.987**	**0.956**	**0.903**	**1.000**	**0.917**

Bold: The parameter setting used in the manuscript.

Table S10 The comparison between PN-DSN and ATSD-DN with different thresholds of non-overlapping ratios (NOR) on the time-series dataset

Method	Parameter setting	N *vs.* M	HCC *vs.* non-HCC		H *vs.* CIR	
		Training set	Training set	Test set	Training set	Test set
PB-DSN		0.898	0.954	0.948	0.966	0.972
ATSD-DN	**0.7**	**0.699**	**0.808**	**0.965**	**0.776**	**0.870**
	0.75	0.699	0.808	0.965	0.776	0.870
	0.8	0.699	0.808	0.965	0.776	0.870

Bold: The parameter setting used in the manuscript.

Table S11 The comparison between PN-DSN and BioNet with different thresholds of false discovery rates (FDR) on the time-series dataset

Method	Parameter setting	N *vs.* M	HCC *vs.* non-HCC		H *vs.* CIR	
		Training set	Training set	Test set	Training set	Test set
PB-DSN		0.898	0.954	0.948	0.966	0.972
BioNet	E-07	0.915	0.934	0.917	0.959	0.889
	5E-07	0.915	0.934	0.917	0.959	0.889
	**E-06**	**0.915**	**0.934**	**0.917**	**0.959**	**0.889**

Bold: The parameter setting used in the manuscript.

Table S12 The comparison between log-fold change of 2 and log-fold change of 3 in PB-DSN on the static dataset

Parameter setting	Number of retained features	EWS *vs.* non-EWS		BL *vs.* non-BL		RMS *vs.* non-RMS		NB *vs.* non-NB	
		Training set	Test set	Training set	Test set	Training set	Test set	Training set	Test set
**|log(fold-change)| =3**	**81**	**1.000**	**1.000**	**1.000**	**1.000**	**0.965**	**1.000**	**1.000**	**1.000**
|log(fold-change)| =2	254	0.940	0.774	1.000	1.000	0.973	1.000	0.941	1.000

Bold: The parameter setting used in the manuscript.

Table S13 The influence of the threshold of *PCC* on the performance of PB-DSN on the static dataset

Parameter setting	EWS vs. non-EWS		BL vs. non-BL		RMS vs. non-RMS		NB vs. non-NB	
	Training set	Test set	Training set	Test set	Training set	Test set	Training set	Test set
0.5	0.878	0.857	1.000	1.000	0.708	0.880	1.000	1.000
0.6	0.996	1.000	1.000	1.000	0.921	1.000	1.000	1.000
**0.7**	**1.000**	**1.000**	**1.000**	**1.000**	**0.965**	**1.000**	**1.000**	**1.000**
0.8	0.975	1.000	0.895	1.000	0.934	1.000	0.845	0.786
0.9	0.953	1.000	0.964	1.000	0.935	1.000	0.685	0.810

Bold: The parameter setting used in the manuscript.

Table S14 The influence of the threshold of *PCC* on the performance of PB-DSN on the time-series dataset

Parameter setting	N *vs.* M	HCC *vs.* non-HCC		H *vs.* CIR	
	Training set	Training set	Test set	Training set	Test set
0.5	0.766	0.639	0.774	0.517	0.833
0.6	0.924	0.923	0.903	0.748	0.824
**0.7**	**0.898**	**0.954**	**0.948**	**0.966**	**0.972**
0.8	0.913	0.685	0.656	0.639	0.611
0.9	0.843	0.878	0.889	0.741	0.935

Bold: The parameter setting used in the manuscript.

Reference

[1] Khan, J. *et al.* Classification and diagnostic prediction of cancers using gene expression profiling and artificial neural networks. *Nat. Med.* 7, 673-679 (2001).

[2] Zeng, J. *et al.* Metabolomics identifies biomarker pattern for early diagnosis of hepatocellular carcinoma: from diethylnitrosamine treated rats to patients. *Sci. Rep.* 5, (2015).
